# Surveillance of severe acute respiratory infections using ICD-10 diagnosis codes and national electronic health records, Denmark, 2022 to 2024

**DOI:** 10.2807/1560-7917.ES.2025.30.33.2400801

**Published:** 2025-08-21

**Authors:** Frederikke Kristensen Lomholt, Karina Lauenborg Møller, Jens Nielsen, Palle Valentiner-Branth, Lasse Skafte Vestergaard

**Affiliations:** 1 Department of Infectious Disease Epidemiology and Prevention, Statens Serum Institut, Copenhagen, Denmark; 2 Division of Disease Preparedness, Statens Serum Institut, Copenhagen, Denmark

**Keywords:** automated surveillance, severe acute respiratory infections (SARI), electronic health records (EHR), register-based, epidemiology, integrated surveillance, case definition

## Abstract

**BACKGROUND:**

The COVID-19 pandemic underscored the need and value of a standardised and timely surveillance system for severe acute respiratory infections (SARI) to inform epidemic preparedness and response.

**AIM:**

We aimed to develop an automated SARI surveillance system using electronic health records retrieved from pre-existing national health registers in Denmark.

**METHODS:**

We used the Danish Civil Register, the Danish National Patient Register and the Danish Microbiology Database to set up the system. First, we determined a SARI case definition for surveillance, choosing among six different potentially usable combinations of ICD-10 diagnosis codes by exploring how each combination captured patient characteristics (age, hospital admission length, mortality, laboratory tests and seasonality). Second, using this case definition, we evaluated the surveillance system’s timeliness and completeness by comparing weekly data reported with a delay of 1, 8, 15, 22 and 29 days, respectively, against a complete set of data extracted after 120 days.

**RESULTS:**

The selected case definition combined ICD-10 codes for influenza (J09-J11), acute lower viral and bacterial respiratory tract infections and bronchiolitis (J12-J22) and COVID-19 (B342A and B972A). With regards to timeliness and completeness of this definition, weekly data reported with a delay of 8 days was 89–93% complete and showed very similar patterns in weekly changes in SARI cases as data reported after 120 days.

**CONCLUSION:**

Our SARI surveillance system detected fluctuations in weekly SARI cases in a consistent and timely manner. We recommend countries to explore using electronic health registers as a resource-efficient alternative to standard SARI sentinel surveillance.

**Key public health message**

**What did you want to address in this study and why?**

The COVID-19 pandemic underscored the need for a surveillance system that timely detects severe acute respiratory infections (SARI) to inform preparedness and response and to evaluate implemented public health measures. Such a surveillance system must be resource-efficient and easy to manage. We hence developed and piloted an automated surveillance system using electronic health records retrieved from pre-existing national health registers in Denmark.

**What have we learnt from this study?**

We found that our new automated Danish SARI surveillance system based on electronic national health records and building on standard ICD-10 diagnosis codes was robust and timely. Furthermore, it accurately identified changes in hospital admissions related to the three common respiratory viruses: severe acute respiratory syndrome coronavirus 2 (SARS-CoV-2), influenza virus and respiratory syncytial virus (RSV).

**What are the implications of your findings for public health?**

The developed SARI surveillance system has now been integrated into the routine weekly surveillance of respiratory infections in Denmark, with output reports published every week. The integrated surveillance of SARI allows for a direct comparison of the burden of severe respiratory infections. Further, the flexibility of the system allows us to add other respiratory infections, and also incorporate data from other health registers, e.g. for vaccine effectiveness evaluation.

## Introduction

Severe acute respiratory infections (SARI) remain a common reason for admission to Danish hospitals, typically caused by a wide range of viral or bacterial seasonal infections. During the COVID-19 pandemic, the number of SARI admissions were largely caused by infections with severe acute respiratory syndrome coronavirus 2 (SARS-CoV-2), while the circulation of other respiratory viruses and bacteria such as influenza virus, respiratory syncytial virus (RSV), *Streptococcus pneumoniae* and *Mycoplasma pneumoniae* decreased markedly as a result of introducing non-pharmaceutical interventions (NPIs) to try to control the spread of COVID-19 [1,2]. As NPIs were lifted, many seasonal respiratory pathogens returned, some with unusual timing and magnitude, underscoring the need for integrated surveillance systems to capture multiple pathogens at the same time [3-7].

There are several surveillance systems in Denmark to monitor the spread of respiratory diseases in the population, such as the syndromic surveillance of influenza-like illness (ILI) through the sentinel surveillance system at general practitioner level [8], and the web-based surveillance based on self-reporting of influenza-like illness in the general population, Influmeter [9], both of which predominantly capture milder infections. Further, using data from the Danish Microbiology Database (MiBa), Denmark has developed pathogen-specific surveillance systems for several respiratory pathogens including influenza virus, RSV and SARS-CoV-2 [10]. These surveillance systems combine results from microbiological tests with clinical information, hospitalisation data and demographic data. However, a national standardised system for syndromic surveillance of SARI that combines surveillance data of severe respiratory cases, with or without data on detected relevant pathogens, has not yet been established. Indeed, the COVID-19 pandemic underscored the need for such a standardised and timely surveillance system in Denmark to quickly detect trends and the spread of both identified and unidentified pathogens, including novel disease. Such detection would allow for timely assessment of respiratory infection severity through a uniform case definition and evaluate the effect of implemented public health interventions.

A severe acute respiratory infection is a clinical diagnosis defined by the World Health Organization (WHO) as an acute respiratory infection with history of fever or measured fever of ≥ 38 C° and cough, with onset within the last 10 days and which requires hospitalisation [11]. The surveillance of trends in SARI cases has traditionally relied on manual case ascertainment reported by sentinel hospitals [12,13], but such a manual system has never been set up for routine surveillance in Denmark. Manual surveillance systems are, however, resource-intensive to operate on a daily routine level and are especially burdensome for healthcare staff. As an alternative approach, we aimed to develop an automated national SARI surveillance system, building on electronic health records (EHR) from pre-existing national registers by applying a ‘proxy’ SARI case definition based on diagnosis codes from the International Statistical Classification of Diseases and Related Health Problems 10th Revision (ICD-10) [14]. Several other European countries are in the process of developing similar EHR-based surveillance systems; however, the optimal case definition remains to be identified [15-17].

In this study, we describe the development and set up of our EHR-based Danish surveillance system for capturing SARI. This includes a review of different combinations of ICD-10 diagnosis codes to identify patients hospitalised with a respiratory infection; the specifications of applied data sources; and a description of linkage algorithms. To evaluate the performance of the surveillance system, we performed analyses on completeness and timeliness of detecting changes in national SARI case load over time.

## Methods

### Data sources

**Figure 1:**
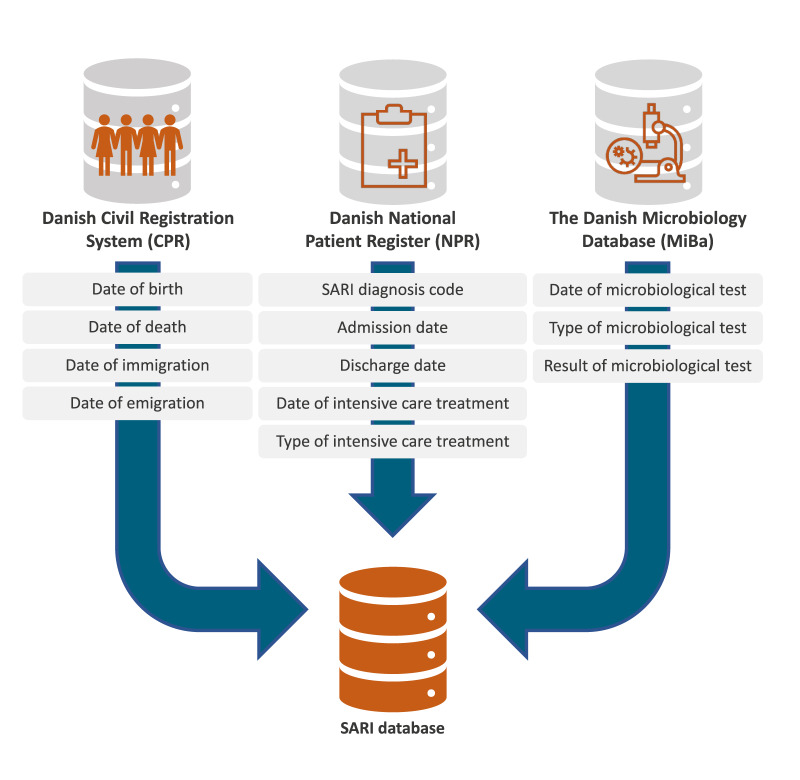
Electronic health records retrieved from national registers to create the national electronic health record-based severe acute respiratory infection surveillance database, Denmark, 2022–2023 (n = 3 registers)

We used three registers for the Danish EHR-based SARI surveillance system: the Danish National Patient Register (NPR) [18]; MiBa [10,19]; and the Danish Civil Registration System (CPR register) [20], see Figure 1. All data were linked on an individual level using the 10-digit civil registration number provided for all Danish residents.

SARI: severe acute respiratory infection.

The NPR holds information on all patient hospital contacts with both private and public hospitals, including all outpatient and inpatient contacts, contacts with emergency departments and contacts with psychiatric departments. All patient hospital contacts are registered with a start and end date and time, one primary ‘action’ diagnosis code and one or more optional secondary diagnoses codes from the ICD-10 [14]. If a patient is transferred between hospital departments, a new patient hospital contact is recorded. The NPR also holds information on procedures performed (e.g. non- invasive ventilation) using a national coding system (SKS) [18]. The local version of the NPR available for surveillance is updated daily.

The Danish Microbiology Database is a national register which includes real-time information on all microbiological test results (positive and negative) analysed by hospital departments of clinical microbiology in Denmark. In Denmark, all samples collected in the primary sector (i.e. at general practitioners) and in the secondary sector are analysed by hospital departments of clinical microbiology. Every day, an updated dataset for each of the pathogens SARS-CoV-2, influenza virus and RSV, including all tests performed (positive and negative) is generated. These datasets include nucleic acid-based (molecular) tests but not antigen tests.

The CPR register holds personal information on all Danish residents including their date of birth, date of death, immigration and emigration. The CPR register was used to define the population under surveillance and to retrieve information on vital status for patients identified in the surveillance system. The CPR register is also updated daily.

### Set-up

Since a new patient hospital contact is recorded when a patient is transferred between hospital departments, a patient can be recorded with several sequential contacts during the same course of hospital admission. To ensure that each patient was only counted a single time for each admission, contacts were merged into unified courses of contacts, hereafter named ‘admissions’. Contacts were merged when a subsequent contact was recorded as started within 4 hours of the previous contact [21]. Consequently, since diagnosis codes are provided for each hospital contact, a patient can be registered with several primary and secondary diagnosis codes during the same admission. For our surveillance system, we included patients with an admission of at least 12 hours, which is the standard definition in other Danish surveillance systems.

We identified potential ICD-10 diagnosis codes to be included as our proxy SARI case definition based on literature review, see Table 1. From the NPR we extracted all recorded hospital admissions between week 1 2022 and week 52 2023 recorded with at least one primary or secondary diagnosis code from Table 1. To select codes to be included in our final case definition, we created six case definitions, based on various combinations of relevant diagnosis codes. Our main case definition (CD1) included all admissions with a diagnosis code of influenza or pneumonia (J09 to J18) or COVID-19 (B342A and B972A). For the second case definition (CD2), we identified additional cases with other lower respiratory tract infections (J20 to J22) not already identified in CD1. For our third case definition (CD3) we identified additional cases with a diagnosis code of an acute upper respiratory tract infection (J00 to J06), while for our fourth case definition (CD4) we identified cases with a diagnosis code of chronic obstructive pulmonary disease (COPD) with lower respiratory tract infection or exacerbation (J44.0 and J44.1). Our fifth case definition (CD5) included cases with adult respiratory distress syndrome (J80) and our sixth case definition (CD6) included cases with respiratory failure (J96). Cases with more than one diagnosis code during the same admission were only included in the case definition with the highest rank, CD1 being highest and CD6 being lowest, i.e. an episode with both diagnosis code J09 and J00 would therefore be included in CD1 and not in CD3.

**Table 1:** The International Statistical Classification of Diseases and Related Health Problems 10th Revision (ICD-10) diagnosis codes [14] evaluated for the identification of severe acute respiratory infection cases grouped by case definition, Denmark, 2022–2023 (n = 6 case definitions)

Diagnosis group	ICD-10 codes	Case definition
**Influenza and pneumonia**	J09: Influenza due to identified zoonotic or pandemic influenza virus	CD1
	J10: Influenza due to identified seasonal influenza virus	
	J11: Influenza, virus not identified	
	J12: Viral pneumonia, not elsewhere classified	
	J13: Pneumonia due to *Streptococcus pneumoniae*	
	J14: Pneumonia due to *Haemophilus influenzae*	
	J15: Bacterial pneumonia, not elsewhere classified	
	J16: Pneumonia due to other infectious organisms, not elsewhere classified	
	J17: Pneumonia in diseases classified elsewhere	
	J18: Pneumonia, organism unspecified	
**COVID-19**	B972A: COVID-19 severe acute respiratory syndrome	
	B342A: COVID-19, not elsewhere classified	
**Other acute lower respiratory tract infections**	J20: Acute bronchitis	CD2
	J21: Acute bronchiolitis	
	J22: Unspecified acute lower respiratory tract infection	
**Acute upper respiratory infections**	J00: Acute nasopharyngitis	CD3
	J01: Acute sinusitis	
	J02: Acute pharyngitis	
	J03: Acute tonsillitis	
	J04: Acute laryngitis and tracheitis	
	J05: Acute obstructive laryngitis [croup] and epiglottitis	
	J06: Acute upper respiratory infections of multiple and unspecified sites	
**Chronic lower respiratory disease**	J44.0: Chronic obstructive pulmonary disease with acute lower respiratory infection	CD4
	J44.1: Chronic obstructive pulmonary disease with acute exacerbation, unspecified	
**Other respiratory diseases principally affecting the interstitium**	J80: Adult respiratory distress syndrome	CD5
**Other diseases of the respiratory system**	J96: Respiratory failure	CD6

CD: case definition.

We compared the cases identified by the different case definitions with regard to the following criteria: age (grouped in the following age groups: 0–4, 5–14, 15–29, 30–64, 65–80 and ≥80 years); probability of having a test for SARS-CoV-2, influenza virus or RSV performed, independent of test result (calculated for each pathogen individually as number of tested patients divided by the total number of patients); probability of a laboratory confirmation of SARS-CoV-2, influenza virus or RSV among those tested; length of hospital stay; provision of intensive care treatment; and 30-day mortality. To investigate any seasonal patterns of admissions for each of the evaluated case definitions, we plotted the number of identified patients by week.

To examine how well the case definitions detected changes in the circulation of SARS-CoV-2, influenza virus and RSV, we plotted the weekly number of cases for each of the case definitions with the weekly number of patients admitted with a positive test for SARS-CoV-2, influenza virus or RSV identified in the pathogen-specific surveillance systems [22]. To further examine the ability of our surveillance system to identify peaks in admissions with these three viruses, we calculated the correlation of weekly number of patients identified by our surveillance system with the sum of patients identified in the pathogen-specific surveillance system by calculating Spearman’s rank correlation coefficient with 95% confidence interval (CI). Spearman’s rank correlation coefficients were calculated for each stepwise expansion of the case definition to include more diagnosis codes, i.e. first for CD1 alone, then for CD1 to CD2, CD1 to CD3 and up to CD1 to CD6, both in the total population and by age group.

In addition, we calculated the weekly and overall sensitivities and positive predictive values for detecting patients in the pathogen-specific surveillance. This was also done for each stepwise expansion of the case definition for the total population as well as by age group. Specificities and negative predictive values could not be calculated as the ‘true negatives’ were unknown.

### Definition of surveillance variables

#### Laboratory confirmed cases

For patients tested at least once for SARS-CoV-2, influenza virus or RSV between 10 days before admission date and up to 3 days after admission date, we included one test per pathogen. We included positive tests over negative tests and for tests with the same result, we kept the test closest to admission date.

#### Patients provided with intensive care treatment

As a proxy to identify patients admitted to an intensive care unit, we used procedure codes for intensive care treatment from the NPR, see Table 2. The procedure codes used in the surveillance system developed and piloted during this study are the same as for the pathogen-specific surveillance systems in Denmark [3,23].

**Table 2:** Procedure codes used to define intensive care treatment during hospital admission, Denmark, 2022–2023 (n = 6 intensive care treatment categories)

ICT category	Procedure code	Procedure name
**Intensive care**	NABB	Intensive care therapy
	NABE	Intensive care observation
**Mechanical ventilation**	BGDA0	Mechanical ventilation
	BGFC32	Continuous positive airway pressure treatment
	BGFC33	Continuous positive airway pressure treatment (intermittent)
**Ventilation**	BGDA1	Non-invasive ventilation
**Dialysis**	BJFD0	Acute dialysis
**ECMO**	BGXA2	Extracorporeal membrane oxygenation (ECMO)
**Heart treatment**	BFHC93	Treatment with vasopressors
	BFHC95	Combination treatment with vasopressors and inotropes

ECMO: extracorporeal membrane oxygenation; ICT: intensive care treatment.

#### Mortality

We used two different measures for mortality, a 30-day mortality identifying all deaths occurring within 30 days from date of admission, including deaths occurring during admission and after discharge, and a 30-day in-hospital mortality including only deaths during admission.

#### Timeliness of reporting and completeness of the surveillance system

For each week from week 40 2023 to week 8 2024, five consecutive weekly data extractions for the database were available. Each Monday, an updated version of the database for the subsequent 5 weeks was created and stored independently. This means that for any given week during this period, the first data were available on Monday in the following week, i.e. 1 day delay, and the last data were available on Monday after 5 weeks, i.e. 29 days delay. After a surveillance period of 4 months, we extracted a final dataset, which we considered complete and appropriate for evaluation.

With week of admission on the x-axis, we plotted the number of cases reported after 1, 8, 15, 22 and 29 days and compared the epidemiological curves against the curve from the complete dataset. For each week, we also calculated the proportion of cases identified at each time point compared to the complete dataset.

All statistical analyses were performed using R software for windows version 4.4.0 [24].

## Results

### Patient characteristics

In the period from 3 January 2022 (Monday in week 1) to 31 December 2023 (Sunday in week 52), a total of 161,323 hospital admissions were identified, of which 113,313 cases were included in CD1, 5,207 cases were included in CD2, 9,588 were included in CD3, 20,353 were included in CD4, 75 were included in CD5 and 1,787 were included in CD6. Table 3 shows the characteristics of patients identified by each case definition.

**Table 3:** Characteristics of patients identified by different combinations of the International Statistical Classification of Diseases and Related Health Problems 10th Revision (ICD-10) diagnosis codes, for case definitions 1 to 6, Denmark, week 1 2022–week 52 2023 (n = 6 case definitions)

Characteristics	Case definition											
	CD1		CD2		CD3		CD4		CD5		CD6	
Total	113,313		5,207		9,588		20,353		75		12,787	
**Age group (years)**	n	%	n	%	n	%	n	%	n	%	n	%
**0–4**	4,048	3.6	3,900	74.9	4,448	46.4	0	0.0	1	1.3	779	6.1
**5–14**	1,012	0.9	107	2.1	910	9.5	1	0.0	0	0.0	377	2.9
**15–29**	4,080	3.6	37	0.7	1,605	16.7	3	0.0	7	9.3	806	6.3
**30–64**	22,619	20.0	295	5.7	1,617	16.9	3,711	18.2	33	44.0	4,091	32.0
**65–79**	39,892	35.2	464	8.9	649	6.8	10,515	51.7	25	33.3	4,545	35.5
**≥80**	41,662	36.8	404	7.8	359	3.7	6,123	30.1	9	12.0	2,189	17.1
**Length of stay (days)**	Median	IQR	Median	(IQR)	Days (n)	Median (IQR)	Days (n)	Median (IQR)	Days (n)	Median (IQR)	Days (n)	Median (IQR)
**Overall**	4.1	2.1–7.2	1.9	1.0–3.9	1.1	0.7–2.2	3.6	1.9–6.1	20.2	8.9–36.0	3.1	1.0–9.3
**Intensive care treatment**	n	%	n	%	n	%	n	%	n	%	n	%
**Patients receiving ICT**	10,520	9.3	778	14.9	1,114	11.6	4,009	19.7	65	86.7	7,315	57.2
**Mortality**	n	%	n	%	n	%	n	%	n	%	n	%
**All mortality**	13,100	11.6	77	1.5	75	0.8	2,033	10.0	29	38.7	2,552	20.0
**In-hospital mortality**	7,667	6.8	30	0.6	43	0.4	1,193	5.9	26	34.7	2,089	16.3
**SARS-CoV-2**	n	%	n	%	n	%	n	%	n	%	n	%
**Tested**	93,227	82.3	3,702	71.1	5,268	54.9	16,014	78.7	46	61.3	5,718	44.7
**Positive** **(n and % of tested)**	30,001	32.2	77	2.1	265	5.0	373	2.3	2	4.3	194	3.4
**Influenza virus**	n	%	n	%	n	%	n	%	n	%	n	%
**Tested**	79,275	70.0	3,935	75.6	4,966	51.8	14,521	71.3	40	53.3	4,424	34.6
**Positive** **(n and % of tested)**	7,429	9.4	19	0.5	127	2.6	174	1.2	1	2.5	39	0.9
**RSV**	n	%	n	%	n	%	n	%	n	%	n	%
**Tested**	40,771	36.0	3,784	72.7	3,838	40.0	6,976	34.3	22	29.3	2,161	16.9
**Positive,** **(n and % of tested)**	2,156	5.3	1,828	48.3	415	10.8	337	4.8	0	0.0	181	8.4

CD: case definition; ICT: intensive care treatment; IQR: interquartile range, RSV: respiratory syncytial virus.

#### Age distribution

For CD1, more than 70% of patients were 65 years or older, whereas patients identified in CD2 (J20-J22) and CD3 (J00-J06) were younger, with more than 70% of patients aged 0–4 years in CD2 and more than 70% younger than 30 years in CD3. Patients identified in CD4 (J44.0 and J44.1) were older with more than 80% over 65 years. The majority of patients identified in CD5 (J80) and CD6 (J96) were aged between 30 and 79 years.

#### Length of stay

Admissions identified in CD1 had a median length of stay in hospital of 4.1 days, while admissions in CD2 and CD3 had shorter length of stay with medians of 1.9 and 1.1 days, respectively. Admissions included in CD4 had a median length of stay of 3.6 days, while the longest length of stay was seen for admissions in CD5 (median 20.2 days).

#### Mortality and intensive care treatment

In-hospital mortality was highest among patients identified in CD5 and CD6, at 34.7% and 16.3%, respectively, compared with 6.8% among patients identified in CD1. In-hospital mortality was low for patients in CD2 and CD3, at 0.6% and 0.4%, respectively. Intensive care treatment was provided for 9.8% of patients in CD1. This proportion was higher in all other case definitions and highest for patients in CD5 and CD6.

#### Laboratory testing

From the laboratory testing, we found that 82.3% of patients identified in CD1 were tested for SARS-CoV-2 with a positivity rate of 32.2% among those tested. For the other case definitions, between 44.7% and 78.7% of patients were tested with positivity rates ranging between 2.1% and 5.0%. We found 70.0% of patients in CD1 were tested for influenza virus, with a positivity rate of 9.4%. For the other case definitions, the proportion of patients tested for influenza virus was between 34.6% and 75.6%, with positivity rates ranging from 0.5% to 2.6%. In addition, 36.0% of patients in CD1 were tested for RSV with a positivity rate of 5.3%. For patients in CD2 and CD3, the proportion tested for RSV was higher at 72.7% and 40.0%, respectively, with positivity rates of 48.3% and 10.8%, respectively.

When including all diagnosis codes from CD1 to CD6, a total of 30,912 patients with laboratory-confirmed SARS-CoV-2 were identified. Of these, most were identified in CD1 alone (n = 30,001, 97.1%), see Supplementary Table S1, which shows the number and proportion of laboratory confirmed cases identified by combinations of case definitions. For RSV, a total of 4,917 patients were identified, of which 2,156 (43.8%) were identified in CD1 alone. This number increased to 3,985 (81.0%) when including CD2 and 4,399 (89.5%) when including CD2 and CD3. A total of 7,789 patients with laboratory-confirmed influenza were identified when including diagnoses from CD1 to CD6. Of these, 7,429 (95.4%) were identified in CD1 alone.

#### Seasonal pattern

**Figure 2:**
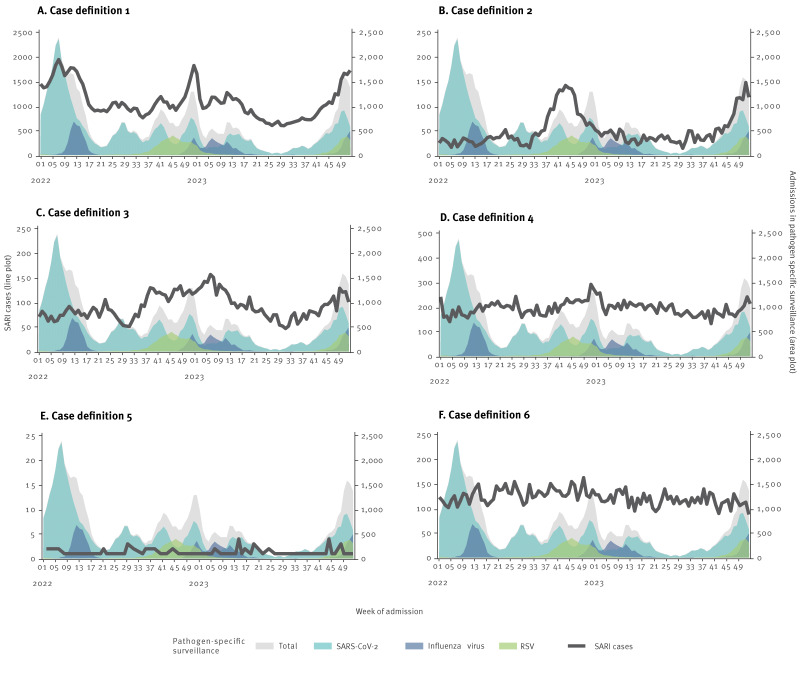
Weekly number of severe acute respiratory infection cases and admissions with severe acute respiratory syndrome coronavirus 2, influenza virus and respiratory syncytial virus separately and combined by case definition, Denmark, week 1 2022–week 52 2023 (n = 6 case definitions)

RSV: respiratory syncytial virus; SARI: severe acute respiratory infection; SARS-CoV-2: severe acute respiratory syndrome coronavirus 2.

Both CD1 and CD2 showed clear seasonal trends in hospital admissions, where CD1 closely followed the combined number of admissions with SARS-CoV-2, influenza virus and RSV from the pathogen-specific surveillance (Figure 2A), while CD2 primarily followed the pattern for admissions with RSV (Figure 2B). There was a less clear seasonal trend seen for CD3. While the number of cases did appear to increase during the winter months (Figure 2C), they did not clearly follow the pattern of any of the three viruses. There did not appear to be a seasonal pattern for CD4 and CD6, and there were too few weekly admissions for CD5 to detect any changes over time. For Spearman’s rank correlation (rs), we calculated the highest correlation for the total population when combining CD1 and CD2 (rs: 0.95, 95% CI: 0.93–0.97). However, the correlation coefficients were very similar for all combinations (Supplementary Table S2 shows rs for different combinations of case definitions). When looking at the correlation by age group, we found that the correlation was highest when combining CD1 and CD2 for children aged 0 to 4 years (rs: 0.89, 95% CI: 0.85–0.93). For the other age groups, the correlation was similar across the different combinations of case definitions.

### Sensitivity and positive predictive value of case definitions

For the weekly sensitivity, we found that during weeks of high activity of RSV, sensitivity of CD1 alone was markedly lower than when adding CD2 (Supplementary Figure S1 shows the weekly sensitivity by combinations of case definitions). We found that, overall, adding CD3 only contributed limited additional sensitivity. Stratified by age group, we found that the decrease in sensitivity for CD1 alone was primarily detected among children aged 0–4 years, while for the other age groups, we found very similar weekly sensitivities for all combinations of case definitions (Supplementary Figure S3 shows the weekly sensitivity by combinations of case definitions stratified by age group). The sensitivity for the entire study period in the total population was 0.58 for CD1, 0.61 for CD1 to CD2 and increasing to 0.64 for CD1 to CD6 (Supplementary Table S3 shows the overall sensitivity and positive predictive value by combinations of case definitions stratified by age group). For children aged 0–4 years, sensitivity for the entire study period was 0.32 for CD1, increasing to 0.57 for CD1 and CD2 and 0.64 for CD1, CD2 and CD3. For the other age groups, expanding the case definition to more than CD1 alone contributed only a limited additional sensitivity.

The positive predictive value was, for all age groups, highest for CD1. For the age groups between 0 to 29 years the positive predictive values dropped only slightly when adding CD2, but dropped more markedly when adding CD3. For the patients aged 30 years and older, the positive predictive values were similar for CD1, CD1 to CD2 and CD1 to CD3, but dropped slightly when adding CD4 to the case definition.

### Assessment of timeliness and completeness of the surveillance system

**Figure 3:**
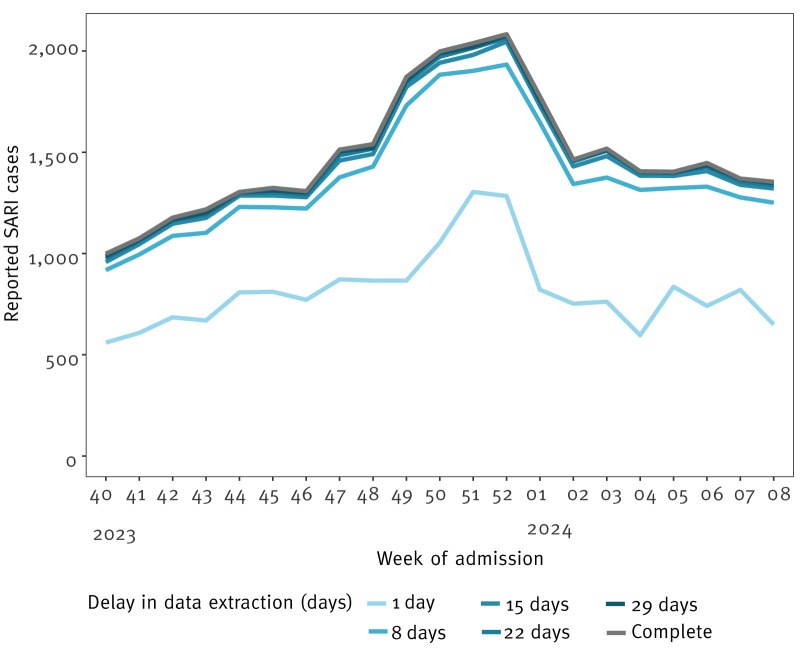
Total number of admissions identified by week when data was extracted with a delay of 1 day, 8 days, 15 days, 22 days and 29 days, respectively, compared with complete dataset, Denmark, week 40 2023–week 8 2024 (n = 6 timepoints)

To assess completeness and timeliness we included patients fulfilling CD1 and CD2. Figure 3 shows the epidemiological curves of weekly number of admissions identified with a delay of 1, 8, 15, 22 and 29 days, respectively, compared to the complete data after 4 months. We found that data retrieved after 8 days or later followed the overall pattern of the complete dataset, while data retrieved after 1 day were less consistent and failed to detect an increase from week 48 to week 49, and the flattening of the curve from week 50 to week 51. Data available with a delay of 1 day appeared more vulnerable to delays in updates from the registers, especially during periods of high case loads.

The minimum weekly completeness for the combined case definition including CD1 and CD2 after 1 day was 41% (week 4 2024) and the maximum weekly completeness was 62% (week 51 2023) compared to the complete data compiled after 4 months. These values increased to 87% and 93%, minimum and maximum values, respectively, after 8 days, and 96% and 99% after 29 days. From the data available after 1 day, between 2% (minimum weekly value) and 5% (maximum weekly value) of patients identified were not found in the final dataset due to later changes in their diagnosis codes related to their admission, removal of a SARI diagnosis or a slight adjustment in admission date. These proportions decreased to 1% and 3%, minimum and maximum weekly values, respectively, at day 8, and 0% and 2% at day 29. The discrepancies between cases identified at day 29 and cases in the complete dataset were largely due slight administrative adjustments in admission date. During the period between week 40 2023 and week 8 2024, the complete data identified a total of 31,189 episodes, while data at day 29 identified a total of 30,990. Of the episodes identified at day 29, only 56 cases were incorrectly identified as SARI, while 255 cases were missing compared to the complete dataset.

## Discussion

The aims of this study were to describe the development and set-up of a new Danish automated register-based SARI surveillance system using electronic health records from existing national registers and to evaluate the reporting timeliness and completeness of this surveillance system. We explored a range of possible ICD-10 diagnosis codes to be included in a proxy SARI case definition based on predefined criteria, and settled on diagnosis codes for influenza (J09-J11), viral and bacterial pneumonia (J12-18), other acute lower respiratory tract infections including bronchiolitis (J20-J22) and COVID-19 (B342 and B972A) to identify SARI cases in our surveillance. We considered CD1 the basic case definition but expanded it to include diagnosis codes J20-J22 (CD2). Even though characteristics of patients in CD1 and CD2 suggested less severe disease (short length of stay and low mortality), by including these diagnosis codes the system would better capture RSV cases and respiratory disease among young children aged 0–4 years. On the other hand, we decided not to include diagnosis codes for upper respiratory tract infections, as these episodes had short length of stay, low mortality and there was no strong seasonal pattern. The additional diagnosis codes from CD4 to CD6 were excluded since these episodes could likely be due to other causes than respiratory infections and including them in the case definition might cause noise to the surveillance.

To further support our decision, we performed analyses of sensitivity overall and by week comparing cases identified when expanding the case definition against cases from the pathogen-specific surveillance systems. We found that especially among children aged 0–4 years, sensitivity increased when expanding the case definition from CD1 to include diagnosis codes from CD1 and CD2, supporting the decision to include these codes. Despite the sensitivity increasing further in this age group when adding diagnosis codes from CD3, the positive predictive value also dropped.

Some other European countries have applied the same set of diagnosis codes, allowing us to draw direct comparison [15,25]. However, since there is no agreed consensus yet on an EHR-based SARI case definition built on diagnosis codes, several alternative options exist to identify SARI cases from electronic health registers. Other countries e.g. Norway and Spain, also include diagnosis codes for upper respiratory tract infections, such as whooping cough and otitis media, in their SARI surveillance systems [13,16,26]. In our analysis, however, we found that upper respiratory tract infections were primarily seen in children and in adults younger than 29 years, causing mild disease with short length of hospital stay. Since the purpose of our surveillance system is to capture cases of severe respiratory infection and to provide an addition to the existing surveillance systems in Denmark, we opted not to include infections from the upper respiratory tract. The consequence of this narrower use of ICD-10 codes is that we may miss respiratory infections among children and young adults, especially for hospital admissions related to RSV. Portugal included other diagnosis codes such as for cardiovascular disease and respiratory symptoms [17]. While codes for cardiovascular disease are associated with the circulation of influenza virus, their association with SARS-CoV-2 is less clear [27]. Further, increasing the number of codes could increase the likelihood that the surveillance system detects considerable increases in respiratory infections, but might, at the same time, detect changes not related to respiratory infections, thereby introducing noise to the surveillance. For the purpose of the Danish surveillance system, where we aim to be able to identify trends in hospital admissions due to respiratory infections, allowing us to evaluate infection severity, we decided to restrict the case definition to more specific diagnosis codes that would still be sensitive enough to detect trends. We also found, in data not shown, that the diagnosis codes J09-J11, B342A and B972A, and J12, J20 and J21 maximised sensitivity and specificity in identifying hospital admissions with laboratory-confirmed infection with influenza virus, SARS-CoV-2 and RSV, respectively [28].

It might not be possible to create a uniform case definition across countries, given differences in coding practices as well as the registers used for setting up register-based systems. Therefore, every country will have to decide for themselves about the most relevant and suitable case definition for their purpose. Meanwhile, exchange of national surveillance results between countries may still be of great public health value to inform about and compare national seasonal trends in respiratory infections.

For the reporting on timeliness and completeness, we found that with a delay of only 8 days, the surveillance system was able to consistently detect changes in hospital admissions with respiratory infections when compared with data extracted after 4 months. A similar level of timeliness has previously been reported for the German SARI surveillance [25]. Data with a delay of 29 days from a 21-week period incorrectly identified only 0.2% of patients and missed only 0.8% of patients when compared to the complete dataset.

We are aware of some limitations of our study. First, we were unable to validate if the patients who we identified through electronic health records would also be identified as SARI cases using the clinical case definition. Since the clinical case definition includes information on symptoms of cough and fever that we are unfortunately not able to capture in the Danish registers it is uncertain as to what proportion of patients actually fulfilled the clinical SARI case definition. In our surveillance system, we rely on the decision of the treating physician providing codes for respiratory infections. Patients identified in the EHR-based surveillance might not strictly follow the clinical case definition, but we aimed to capture hospital admissions with respiratory infections, which is the aim of the clinical SARI case definition. Since we were not able to validate our case definitions against the clinical SARI case definition, we instead compared the case definitions against the trends in three major contributors of respiratory infections, i.e. influenza virus, RSV and SARS-CoV-2. However, other pathogens may have different patterns and we do not know how well the case definition is able to capture these. One study evaluating the positive predictive value of ICD-10 codes for COVID-19 found a positive predictive value of 99% [29] and another study on diagnosis codes for influenza found a positive predictive value of 87.9% [30]. Compared with the pathogen-specific surveillance system, the overall sensitivity was 0.61 and only limited improvements were detected when expanding the case definition with the codes investigated in this study. The relatively low overall sensitivity was primarily driven by a low sensitivity to RSV. One reason for this is low sensitivity is that other codes, not explored in the set-up of this surveillance, may be used to identify hospital admissions with a positive test for RSV. Another reason is that if RSV did not cause respiratory symptoms in a patient, the patient was therefore not coded with a respiratory diagnosis. While pathogen-specific surveillance systems aim to capture anyone admitted with a positive test, the aim of this surveillance system is to capture patients admitted with respiratory symptoms due to a respiratory infection, thus we do not expect a sensitivity of 100%.

Another limitation is that we decided to pool all diagnosis codes for upper respiratory tract infections together (J00-J06) and we were therefore not able to identify whether patients who were admitted with some of these diagnosis codes (i.e. epiglottitis, J05.1) had more severe disease than other patients within CD3.

A further limitation is that diagnosis codes for patient contacts can be updated several times until discharge, and it is not possible to identify diagnosis codes provided at time of admission versus the diagnosis codes used at the time of discharge from hospital. For this study, we used the codes available at the time of data extraction, and as data were collected retrospectively, this study primarily relied on discharge codes. If diagnosis codes at admission were different from those provided later, we may have missed some codes for early case detection. The timeliness analysis did, however, show that data on day 8 were very consistent with data reported later, suggesting that surveillance is possible in a ‘close to real-time´-scenario. Although the timeliness analysis only covers 21 weeks and a single winter season, we believe the results are representative because the database is based on pre-existing national registers. Having reliable data available on day 8 is useful for the evaluation of the immediate severity and impact of current respiratory infections in society. However, for outbreak detection and for guiding pandemic preparedness and the volume of hospital services required, a true real-time surveillance would be needed. An alternative option would be to use early data, even if not complete, to model the expected case load. The possibility of modelling, such as nowcasting, should be explored to further improve the applicability of the surveillance system for pandemic preparedness. It is also important to bear in mind that surveillance of admissions caused by known pathogens will never be as timely as case detection since case detection requires only a positive test and is not delayed by adding additional epidemiological information such as hospital admission. Further, due to the nature of respiratory infections, it is expected that the number of cases will increase before the number of admissions.

## Conclusion

We found that our newly developed Danish SARI surveillance system based on existing national electronic health records served as a stable, timely and resource-efficient surveillance system and an alternative to manual surveillance. Using our proxy SARI case definition with diagnosis codes J09-J22, B342A and B972A made it possible to identify changes in severe acute respiratory infections caused by SARS-CoV-2, influenza virus and RSV. A major strength of the system is the nationwide coverage, including all age groups, and the flexibility of the system allowing for combining data with other national registers to include information, e.g. on vaccination status and chronic medical conditions. The surveillance system may be further strengthened by including additional respiratory pathogens in the future.

## Data Availability

Not applicable.

## Supplementary Data

428000 Supplementary Material
