# Supplementary material for: Surveillance of severe acute respiratory infections using ICD-10 diagnosis codes and national electronic health records, Denmark, 2022 to 2024
Source: Euro Surveill. 2025 Aug 21;30(33):2400801. doi: 10.2807/1560-7917.ES.2025.30.33.2400801 (PMC12372893; doi:10.2807/1560-7917.ES.2025.30.33.2400801)
Supplement: Supplementary Material [file 24-00801_LOMHOLT_Supplement.pdf]

Supplementary

This supplementary material is hosted by *Eurosurveillance* as supporting information alongside the article “Surveillance of severe acute respiratory infections using ICD-10 diagnosis codes and national electronic health records, Denmark, 2022 to 2024”, on behalf of the authors, who remain responsible for the accuracy and appropriateness of the content. The same standards for ethics, copyright, attributions and permissions as for the article apply. Supplements are not edited by *Eurosurveillance* and the journal is not responsible for the maintenance of any links or email addresses provided therein.

Laboratory confirmed SARI cases 2

Supplementary Table 1. Number and proportion of laboratory-confirmed SARS-CoV-2, RSV and influenza cases identified by combinations of case definition. 2

Spearman’s rank correlation 2

Supplementary Table 2. Spearman’s rank coefficient by age group and case definition, 2022-2023. 2

Sensitivity, positive predictive value and clinical utility index 3

Supplementary Table 3. Overall sensitivity, positive predictive value and clinical utility index, 2022-2023 3

Weekly sensitivity and positive predictive value, total population 5

Sup. Figure 1. Weekly sensitivity for identifying individuals admitted with SARS-CoV-2, influenza virus and RSV from the pathogen-specific surveillance grouped by combinations of case definitions, 2022-2023. 5

Sup. Figure 2. Weekly positive predictive value for identifying individuals admitted with SARS-CoV-2, influenza virus and RSV from the pathogen-specific surveillance grouped by combinations of case definitions, 2022-2023 5

Weekly sensitivity and positive predictive value by age group 6

Sup. Figure 3. Weekly sensitivity for identifying individuals admitted with SARS-CoV-2, influenza virus and RSV from the pathogen-specific surveillance grouped by combinations of case definitions, by age group, 2022-2023. 6

Sup. Figure 4. Weekly positive predictive value for identifying individuals admitted with SARS-CoV-2, influenza virus and RSV from the pathogen-specific surveillance grouped by combinations of case definitions, by age group, 2022-2023 6

Laboratory confirmed SARI cases

Table S1. Number and proportion of laboratory-confirmed SARS-CoV-2, RSV and influenza cases identified by combinations of case definition.

Pathogen	Total	Case definition, n (% of total)											
		CD1		CD1-CD2		CD1-CD3		CD1-CD4		CD1-CD5		CD1-CD6	
**SARS-CoV-2**	30 912	30 001	97.1	30 078	97.3	30 343	98.2	30 716	99.4	30 718	99.4	30 912	100.0
**RSV**	4 917	2 156	43.8	3 984	81.0	4 399	89.5	4 736	96.3	4 736	96.3	4 917	100.0
**Influenza**	7 789	7 429	95.4	7 448	95.6	7 575	97.3	7 749	99.5	7 750	99.5	7 789	100.0

Spearman’s rank correlation

Supplementary table 2 shows the correlation between number of weekly SARI cases identified when gradually expanding the case definition to include more diagnosis codes and the combined number of individuals admitted with SARS-CoV-2, influenza virus and RSV in the pathogen-specific surveillance. Spearman’s rank correlation is calculated in total and by age group.

Table S2. Spearman’s rank coefficient by age group and case definition, 2022-2023.

Age group	Case definition	Spearman’s rank coefficient [95% CI]
**0-4**	CD1	0.84 [0.77; 0.89]
**0-4**	CD1-CD2	0.89 [0.85; 0.93]
**0-4**	CD1-CD3	0.85 [0.79; 0.90]
**0-4**	CD1-CD4	0.85 [0.79; 0.90]
**0-4**	CD1-CD5	0.85 [0.79; 0.90]
**0-4**	CD1-CD6	0.85 [0.79; 0.90]
**5-14**	CD1	0.84 [0.77; 0.89]
**5-14**	CD1-CD2	0.68 [0.57; 0.78]
**5-14**	CD1-CD3	0.71 [0.60; 0.79]
**5-14**	CD1-CD4	0.71 [0.60; 0.79]
**5-14**	CD1-CD5	0.71 [0.60; 0.79]
**5-14**	CD1-CD6	0.71 [0.60; 0.80]
**15-29**	CD1	0.80 [0.72; 0.86]
**15-29**	CD1-CD2	0.80 [0.72; 0.86]
**15-29**	CD1-CD3	0.78 [0.69; 0.85]
**15-29**	CD1-CD4	0.78 [0.69; 0.85]
**15-29**	CD1-CD5	0.78 [0.69; 0.84]
**15-29**	CD1-CD6	0.77 [0.68; 0.84]
**30-64**	CD1	0.91 [0.87; 0.94]
**30-64**	CD1-CD2	0.92 [0.88; 0.94]
**30-64**	CD1-CD3	0.92 [0.88; 0.94]
**30-64**	CD1-CD4	0.91 [0.87; 0.94]
**30-64**	CD1-CD5	0.91 [0.88; 0.94]
**30-64**	CD1-CD6	0.91 [0.87; 0.94]
**65-79**	CD1	0.92 [0.89; 0.95]

**65-79**	CD1-CD2	0.92 [0.88; 0.94]
**65-79**	CD1-CD3	0.92 [0.88; 0.94]
**65-79**	CD1-CD4	0.89 [0.84; 0.92]
**65-79**	CD1-CD5	0.89 [0.84; 0.92]
**65-79**	CD1-CD6	0.88 [0.83; 0.92]
**80+**	CD1	0.94 [0.91; 0.96]
**80+**	CD1-CD2	0.94 [0.91; 0.96]
**80+**	CD1-CD3	0.94 [0.91; 0.96]
**80+**	CD1-CD4	0.93 [0.90; 0.95]
**80+**	CD1-CD5	0.93 [0.90; 0.95]
**80+**	CD1-CD6	0.94 [0.91; 0.96]
**Total**	CD1	0.93 [0.90; 0.96]
**Total**	CD1-CD2	0.95 [0.93; 0.97]
**Total**	CD1-CD3	0.94 [0.92; 0.96]
**Total**	CD1-CD4	0.94 [0.91; 0.96]
**Total**	CD1-CD5	0.94 [0.91; 0.96]
**Total**	CD1-CD6	0.93 [0.90; 0.95]

Sensitivity, positive predictive value and clinical utility index

In the following tables and plots, we have calculated the sensitivity, positive predictive value and clinical utility index for identifying individuals in the pathogen-specific surveillance admitted with SARS-CoV-2, influenza or RSV. The metrics were calculated by consecutively adding groups of diagnosis codes from the six distinct case definition starting with only CD1.

Table S3. Overall sensitivity, positive predictive value and clinical utility index, 2022-2023

Age group	Case definition	Sensitivity	Positive predictive value (PPV)	Clinical utility index (sensitivity * PPV)
**0-4**	CD1	0.32	0.55	0.18
**0-4**	CD1-CD2	0.57	0.49	0.28
**0-4**	CD1-CD3	0.64	0.36	0.23
**0-4**	CD1-CD4	0.64	0.36	0.23
**0-4**	CD1-CD5	0.64	0.36	0.23
**0-4**	CD1-CD6	0.66	0.35	0.23
**5-14**	CD1	0.41	0.49	0.20
**5-14**	CD1-CD2	0.42	0.45	0.19
**5-14**	CD1-CD3	0.46	0.28	0.13
**5-14**	CD1-CD4	0.46	0.28	0.13
**5-14**	CD1-CD5	0.46	0.28	0.13
**5-14**	CD1-CD6	0.47	0.24	0.11
**15-29**	CD1	0.40	0.36	0.14
**15-29**	CD1-CD2	0.40	0.36	0.14

**15-29**	CD1-CD3	0.41	0.26	0.11
**15-29**	CD1-CD4	0.41	0.26	0.11
**15-29**	CD1-CD5	0.41	0.26	0.11
**15-29**	CD1-CD6	0.41	0.23	0.10
**30-64**	CD1	0.51	0.35	0.18
**30-64**	CD1-CD2	0.51	0.35	0.18
**30-64**	CD1-CD3	0.52	0.33	0.17
**30-64**	CD1-CD4	0.52	0.29	0.15
**30-64**	CD1-CD5	0.52	0.29	0.15
**30-64**	CD1-CD6	0.53	0.25	0.13
**65-79**	CD1	0.64	0.33	0.21
**65-79**	CD1-CD2	0.65	0.33	0.21
**65-79**	CD1-CD3	0.65	0.33	0.21
**65-79**	CD1-CD4	0.67	0.27	0.18
**65-79**	CD1-CD5	0.67	0.27	0.18
**65-79**	CD1-CD6	0.67	0.25	0.17
**80+**	CD1	0.72	0.33	0.23
**80+**	CD1-CD2	0.72	0.33	0.23
**80+**	CD1-CD3	0.72	0.32	0.23
**80+**	CD1-CD4	0.73	0.29	0.21
**80+**	CD1-CD5	0.73	0.29	0.21
**80+**	CD1-CD6	0.74	0.28	0.20
**Total**	CD1	0.58	0.34	0.20
**Total**	CD1-CD2	0.61	0.34	0.21
**Total**	CD1-CD3	0.62	0.32	0.20
**Total**	CD1-CD4	0.63	0.29	0.18
**Total**	CD1-CD5	0.63	0.29	0.18
**Total**	CD1-CD6	0.64	0.27	0.17

Weekly sensitivity and positive predictive value, total population

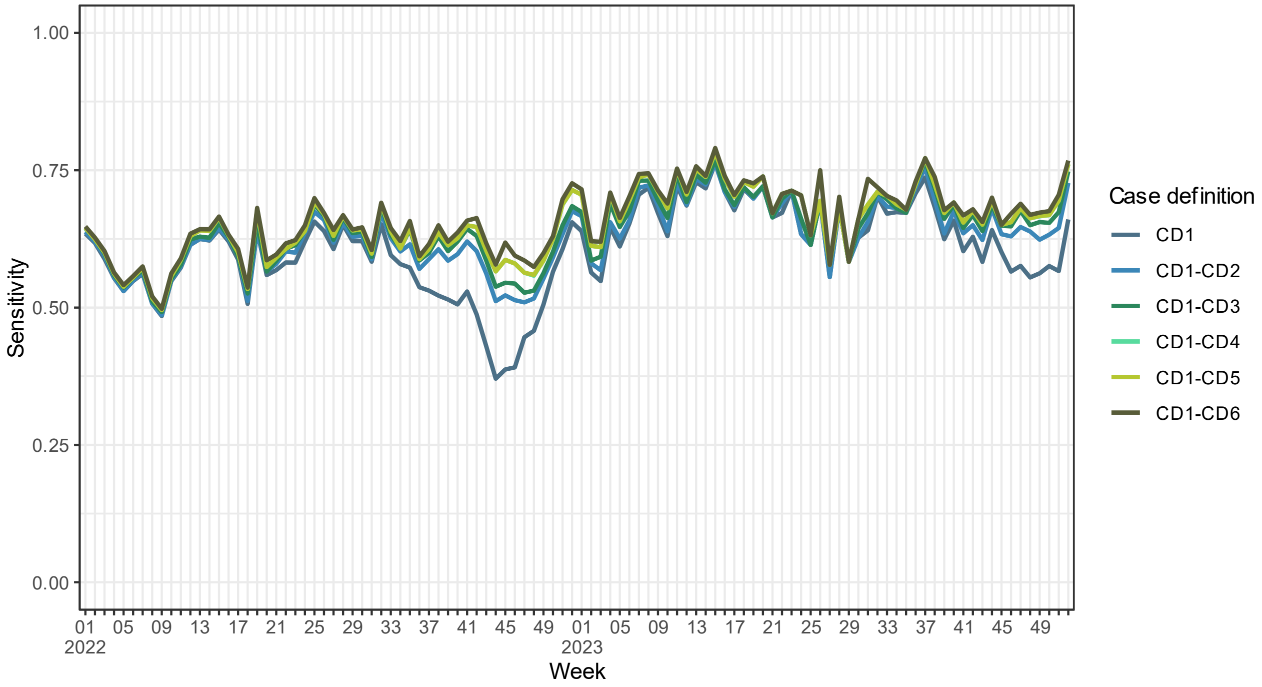

Figure S1. Weekly sensitivity for identifying individuals admitted with SARS-CoV-2, influenza virus and RSV from the pathogen-specific surveillance grouped by combinations of case definitions, 2022-2023.

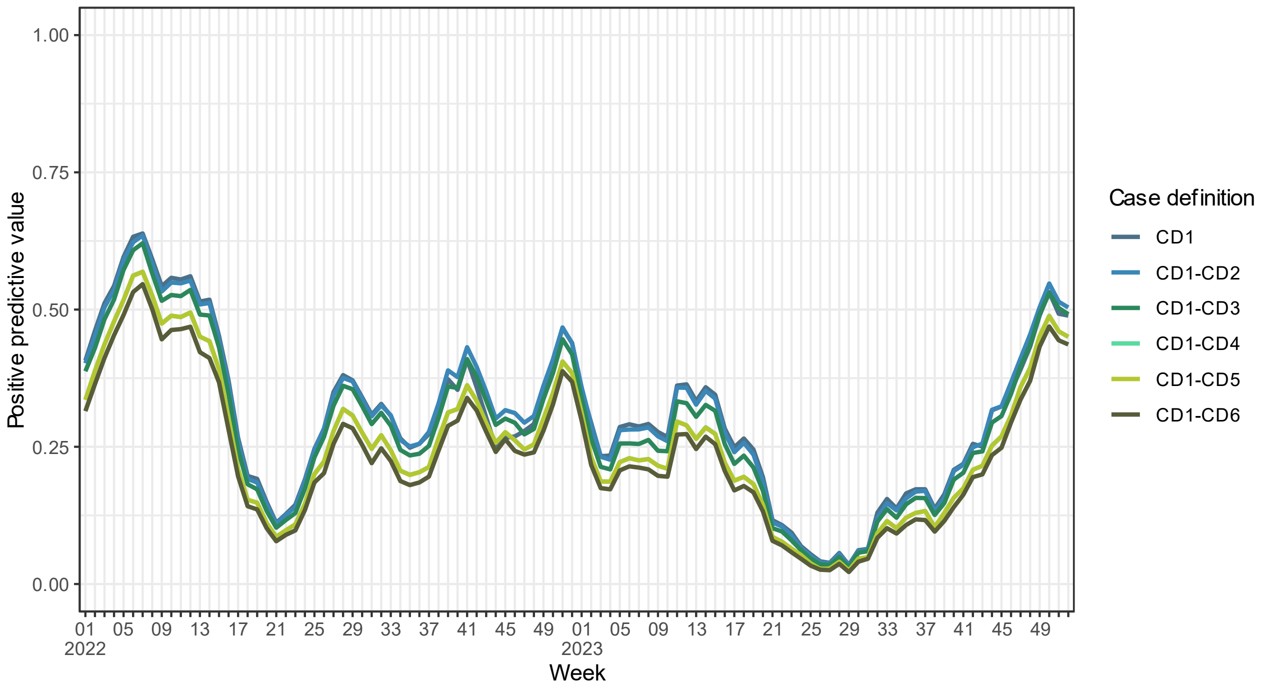

Figure S2. Weekly positive predictive value for identifying individuals admitted with SARS-CoV-2, influenza virus and RSV from the pathogen-specific surveillance grouped by combinations of case definitions, 2022-2023.

Weekly sensitivity and positive predictive value by age group

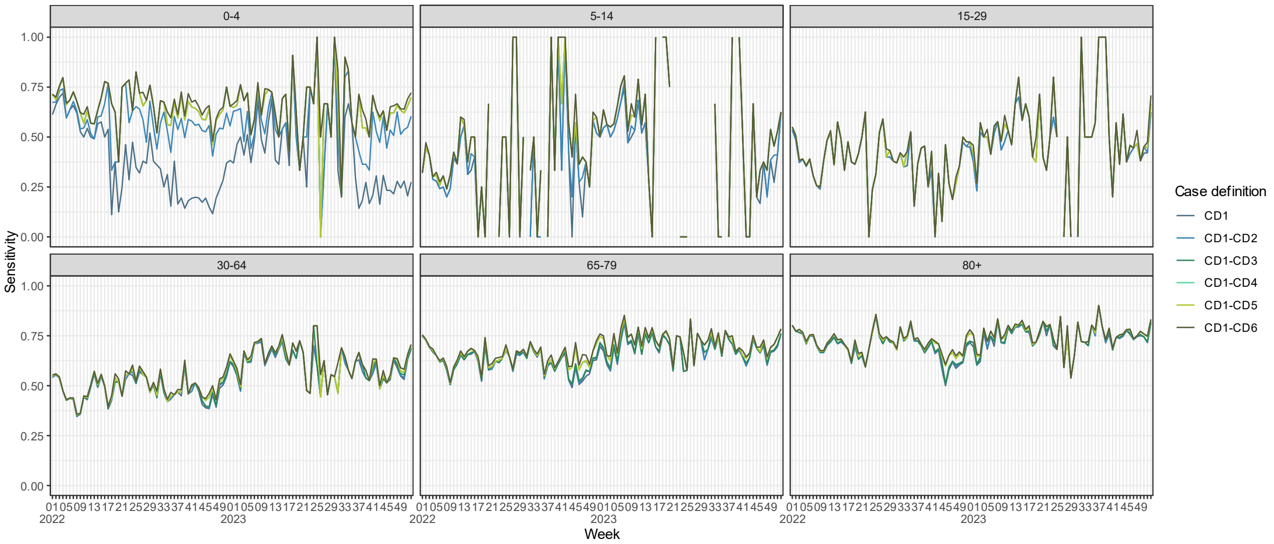

Figure S3. Weekly sensitivity for identifying individuals admitted with SARS-CoV-2, influenza virus and RSV from the pathogen-specific surveillance grouped by combinations of case definitions, by age group, 2022-2023.

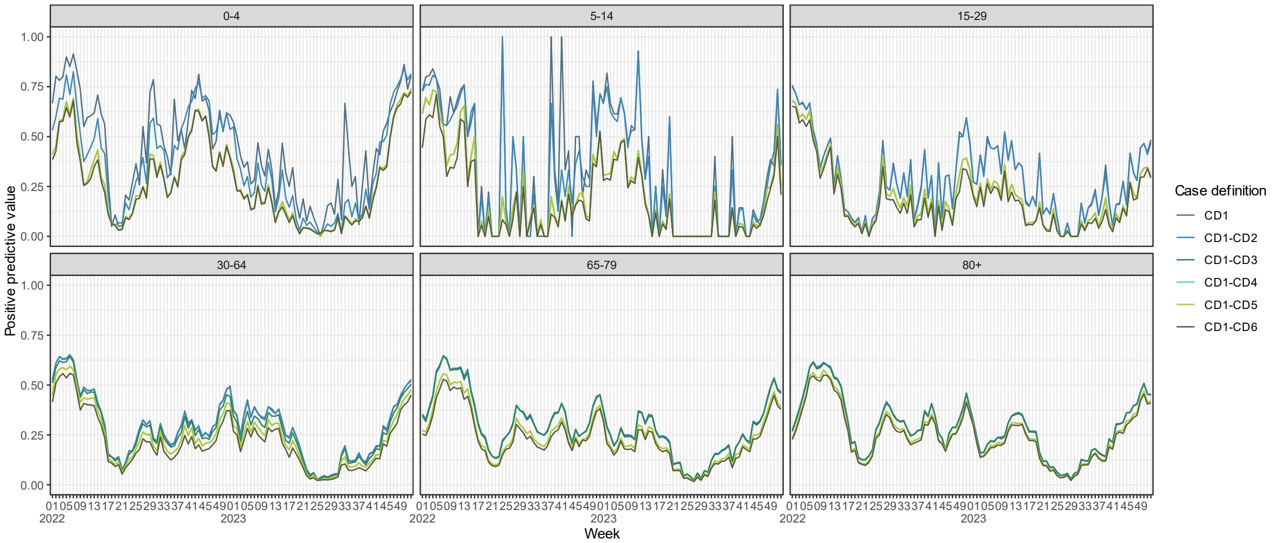

Figure S4. Weekly positive predictive value for identifying individuals admitted with SARS-CoV-2, influenza virus and RSV from the pathogen-specific surveillance grouped by combinations of case definitions, by age group, 2022-2023.
